# Our Original Department

**Published:** 1873-07

**Authors:** 


*Our Original Department.*

We desire to call attention to this department of the present number, which we think will, notwithstanding the room we give it, prove interesting and instructive to our readers. We desire also to express our thanks to our friends, one and all, who have contributed to our pages. By their favors this department of the Record has been made second to no other journal in the country. We again invite our friends to send us articles, and we promise to publish as rapidly as possible.

In the next number we will lay before our readers a valuable article from Dr. Thomas A. Cooke, of Louisiana, on Fever, and publish an address prepared by Drs. J. W. M. Shattuck and W. L. Lipscomb, and read before the Columbus and Lowndes County (Miss.) Medical Society. This address will furnish our readers food for reflection, being a review of the history of medicine and many valuable thoughts on medical ethics. They will find it not only what the profession needs, but what each medical man should delight to "preach from the house-tops" both to the professional and non-professional public. In short, it inculcates truths which, if known by all, both profession and people, would accomplish great good.

